# Perceptions and Opinions Towards Data-Sharing: A Survey of Addiction Journal Editorial Board Members

**DOI:** 10.35122/001c.35597

**Published:** 2022-05-19

**Authors:** J. Michael Anderson, Austin Johnson, Shelby Rauh, Bradley Johnson, Max Bouvette, Isabel Pinero, Jason Beaman, Matt Vassar

**Affiliations:** 1 Center for Health Sciences, Oklahoma State University,; 2 University of Oklahoma

**Keywords:** Data-sharing, editors, addiction, board members, survey

## Abstract

**Background:**

We surveyed addiction journal editorial board members to better understand their opinions towards data-sharing.

**Methods:**

Survey items consisted of Likert-type (e.g., one to five scale), multiple-choice, and free-response questions. Journal websites were searched for names and email addresses. Emails were distributed using SurveyMonkey. Descriptive statistics were used to characterize the responses.

**Results:**

We received 178 responses (of 1039; 17.1%). Of these, 174 individuals agreed to participate in our study (97.8%). Most respondents did not know whether their journal had a data-sharing policy. Board members “somewhat agree” that addiction journals should recommend but not require data-sharing for submitted manuscripts [M=4.09 (SD=0.06); 95% CI: 3.97–4.22]. Items with the highest perceived benefit ratings were “secondary data use (e.g., meta-analysis)” [M=3.44 (SD=0.06); 95% CI: 3.31–3.56] and “increased transparency” [M=3.29 (SD=0.07); 95% CI: 3.14–3.43]. Items perceived to be the greatest barrier to data-sharing included “lack of metadata standards” [M=3.21 (SD=0.08); 95% CI: 3.06–3.36], “no incentive” [M=3.43 (SD=0.07); 95% CI: 3.30–3.57], “inadequate resources” [M=3.53 (SD=0.05); 95% CI: 3.42–3.63], and “protection of privacy”[M=3.22 (SD=0.07); 95% CI: 3.07–3.36].

**Conclusion:**

Our results suggest addiction journal editorial board members believe data-sharing has a level of importance within the research community. However, most board members are unaware of their journals’ data-sharing policies, and most data-sharing should be recommended but not required. Future efforts aimed at better understanding common reservations and benefits towards data-sharing, as well as avenues to optimize data-sharing while minimizing potential risks, are warranted.

## INTRODUCTION

Globally, upwards of US$240 billion is spent on biomedical and health research each year.^1^ However, current estimates suggest that roughly 85% of research funding, equating to more than US$200 billion, is potentially wasted.^2^ Several key factors contributing to this immense research waste include poor research question selection, inadequate study design, poor reporting, and selective non-publication of results.^2^ Although significant advances in medicine have been made, some have argued that the potential for greater improvements in medical outcomes is possible if the inadequacies and shortcomings of the ways in which biomedical research is conducted and reported can be addressed.^3^ While efforts to better the quality of biomedical research have been made,^4–6^ the optimum framework to elicit significant change is still subject to debate. One solution to increase the quality and transparency of biomedical research, while simultaneously helping to mitigate the extent of research waste, is through sharing of complete data sets.^7–9^

Efforts to increase data-sharing practices have been the focus of many governmental and research organizations. For example, the National Institutes of Health (NIH), the world’s largest funder of biomedical research, is currently working to unify a data-sharing policy.^10^ Beginning in 2013, all U.S. federally-funded research agencies receiving more than US$100 million in annual research funds were required to develop a systematic process to make results from publicly-funded research initiatives freely and easily accessible.^11^ Similarly, the expectations placed on clinical trialists by health-research funders, biomedical journals, and other regulated entities to publicly share research data are becoming more common within the medical community.^12–14^ Initiatives pioneered by groups such as the European Medicine Agency and the National Academies of Sciences, Engineering, and Medicine have shifted the conversation surrounding data-sharing from one that simply promotes open data-sharing practices to one that contends sharing de-identified human research data should be a routine, mandatory aspect of clinical research.^15–17^ Despite increasing attention towards and support of data-sharing policies, participation in data-sharing among biomedical researchers remains low.^18^ For instance, a 2019 cross-sectional analysis published in *Addictive Behaviors* found that among nearly four hundred randomized controlled trials focused on addiction interventions, none shared their data publicly.^19^ Failing to provide access to clinical trial data may have implications for the scientific community; however, barriers that may hinder or prevent one from sharing their data altogether must be considered.

Perceived barriers surrounding data-sharing have contributed to hesitancies in adopting a universal data-sharing policy. One example is the financial feasibility and sustainability of data-sharing.^20^ Other researchers may be reluctant to share their data for fear of inadvertent, or deliberate, misrepresentation of datasets, or for fear that other groups may attempt to disprove the findings of their original study.^21^ Further, the heterogeneity among current data-sharing policies governing what, when, where, and how data must be shared adds to the complexity of the expectations placed on researchers. Even though these perceived barriers exist, the potential benefits of sharing clinical trial data transcend throughout the medical community.^8,9,22^

Sharing clinical trial data may serve to increase the transparency and reproducibility of medical research.^8,9,22^ Additionally, by making de-identified human trial data available, research participants’ valuable contribution to the advancement of medical science may be respected while allowing for a “natural extension of the collaboration that is common in the scientific community”.^23^ A 2015 report published by the Institutes of Medicine (IOM) argues the benefits of sharing trial data may increase the public’s trust in the research process by permitting verification of original findings.^24^ The National Addiction and HIV Data Archive Program has implemented practices to “acquire, preserve, and disseminate data relevant to drug addiction and HIV research,” thereby fostering the opportunity for researchers to reproduce study findings or conduct novel studies that answer different, yet important, questions.^25^ Similarly, the Health Insurance Portability and Accountability Act (HIPAA) Privacy Rule 45 C.F.R. § 164.514(e) encourages the use of “limited data sets”—data sets that have been “de-identified” but not completely “anonymized,” as pertinent information such as birthdates, dates of treatment, and certain geographic data are often necessary for public health research efforts—as a means of scientific advancement while ensuring a patient’s right to privacy.^17^ Another prominent entity in the field of addiction medicine with strong opinions towards data sharing is the National Institute on Drug Abuse (NIDA). NIDA has stated that sharing clinical trial data is necessary to “lead the Nation in bringing the power of science to bear on drug abuse and addiction.”^26^ Other perceived benefits include increased rigor and transparency of study designs and outcomes, allowing for more informed patient and clinician decision-making and potentially mitigating negative outcomes that correspond with the collection of human subject data in future research projects.^6,23^ Even though there appears to be increasing support for widespread data-sharing, the barriers and benefits may differ between research stakeholders.

The perceived risks and benefits for sharing de-identified human trial data may vary among those in the research community depending on what each stakeholder (e.g., funding agencies, medical journals, researchers) serves to gain (or lose). Because opinions from these groups may not completely align, a unified data-sharing policy that would facilitate a simple, streamlined, and efficient process for data-sharing does not exist. To better understand the disconnect and commonalities of opinions regarding the topic of data-sharing, we aimed to determine perceptions, attitudes, and beliefs regarding the endorsement of and resistance to the implementation of data-sharing policies, specifically in the field of addiction medicine, by surveying the editorial board members of top addiction medicine journals.

## MATERIALS AND METHODS

This cross-sectional survey study was approved by the Oklahoma State University Institutional Review Board (IRB) committee (Approval No 2020061). All study materials — including the survey, data sheets, analysis scripts, and protocol — are available on the Open Science Framework (https://osf.io/63jyz/).

### PATIENT AND PUBLIC INVOLVEMENT

No patient involved.

### SURVEY DEVELOPMENT

We adhered to the reporting standards outlined in the Checklist for Reporting of Survey Studies (CROSS).^27^ Survey questions were developed through discussion among team members, as well as through reference to previous investigations into potential factors affecting compliance with data-sharing requests.^28–31^ These investigations provided examples of both potential barriers and benefits to data-sharing, in addition to those suggested by the authors of the present study. Survey questions consisted of multiple question types, including Likert type (e.g., “1 - Strongly Agree” to “5 - Strongly Disagree”), multiple choice, and free response. Several questions were included regarding limitations to data-sharing to observe attitudes towards perceived barriers to data-sharing.^28^ In an attempt to optimize response rates, we consulted a systematic review of factors that have been shown to possibly affect survey response rate.^32^ These factors included the disclosure of NIH funding for completion of the present work,^33,34^ emphasis on brevity,^35^ and use of an established survey platform (SurveyMonkey, a secure web application used for generating and managing online surveys^36^); these factors have been shown to increase response rates.

### SAMPLE SIZE DETERMINATION

We used the power tables in Price et al.^37^ to estimate the sample size for our survey. Specifically, we assumed a 50/50 split in the attitudes toward data-sharing among survey participants and a 5% sampling error. Based on these assumptions—and to generalize our survey to a group of one thousand addiction medicine journal editorial board members in positions of authority who may be capable of implementing actionable data-sharing policies—we estimated a response rate of 278.

### SURVEY DISTRIBUTION

We included the top twenty addiction medicine journals, according to Google Scholar Metrics. This list of journals can be found in Supplemental Table 1. We searched for an email address for each potential respondent on the journal’s website. All emails for eligible board members were collected from November 12, 2020, to June 1, 2021. One of us (S.R.) distributed the standardized survey to the editorial board members using SurveyMonkey. Emails with a direct link to our survey were sent once per week for three consecutive weeks beginning on June 7, 2021. If no response was received eight weeks after the date of the first email or if the email address was returned as inactive or invalid we considered the editorial board member as non-contactable. This method of email contact has been successfully used in previous studies.^38,39^ Of note, surveys were distributed in a manner such that respondents were anonymously removed from the email list if the respondent completed the survey on one of the prior email queries (i.e., the SurveyMonkey platform would not email editors after the email associated with that individual had logged a response).

### PROTOCOL DEVIATION

A sizable portion of surveys were returned incomplete (e.g., respondents discontinued the survey prior to completion or did not answer pertinent questions while completing the survey). To account for these missing completely at random (MCAR) data points, we performed multiple imputation analyses, which requires multiple copies of a dataset with imputed values substituting the missing values.^40^ The use of multiple imputation analysis was based on its effectiveness at accounting for missing data and due to the flexibility and ease of implementation described elsewhere.^40^ The imputed values were based on the responses to the other survey questions, thereby allowing for a degree of uncertainty in the missing data to be maintained.

### STATISTICAL ANALYSIS

Missing data were handled using multiple imputation analysis for MCAR data. Descriptive statistics—including frequencies, mean, standard deviations (SD), and 95% confidence intervals—were used to characterize the responses of participants. Stata 16.1 (StataCorp, College Station, Texas, USA) was used for all analyses.

## RESULTS

**Figure 1.**
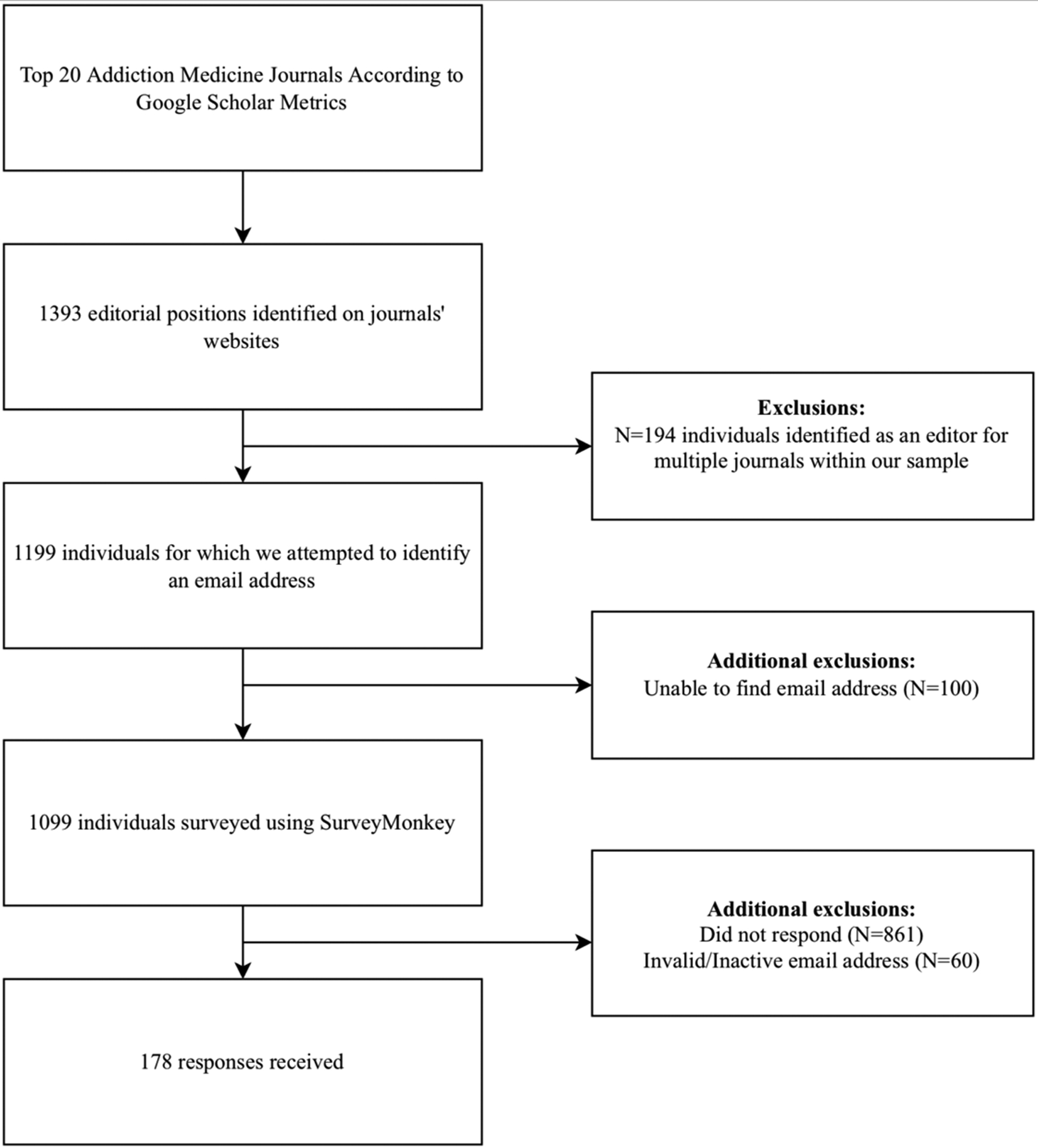
Inclusion and exclusion of survey respondents

We identified 1393 editorial positions among the top twenty addiction medicine journals, according to Google Scholar Metrics. Of these members, 194 (13.9%) held editorial positions at multiple journals in our sample. To prevent duplicate responses, we distributed our survey to these board members as if they held only a single board member position at one journal. We were unable to locate email addresses for one hundred board members (of 1393; 7.2%). Altogether, we successfully identified email addresses for 1099 unique editorial board members (of 1393; 78.9%). The survey was distributed to these 1099 email addresses, of which 60 (5.5%) were returned as invalid and were subsequently excluded from analysis. Our sample comprised 1039 valid email addresses, of which 178 responses were received (17.1%). Of these responses, 174 individuals agreed to participate in our study (97.8%) and 4 elected not to participate (2.2%) (Figure 1). This response rate accords with previously published electronic survey studies in the medical literature.^41,42^

### CHARACTERISTICS OF BOARD MEMBERS

One hundred thirteen respondents (of 174; 64.9%) completed the board member demographic portion of our survey. Sixty-three respondents were male (of 113; 55.8%), forty-eight were female (of 113; 42.5%), and two (of 113; 1.8%) preferred not to respond. Common roles held by respondents at their respective journals included associate editor (65/174; 36.537.4%), consulting editor (8/174; 4.6%), and peer reviewer (7/174; 4.0%), among others. Most respondents indicated they held other positions outside their respective journals, including researcher (83/230; 36.1%) and professor positions (61/230; 26.5%). The number of years spent as an editorial board member varied among respondents, with the majority having worked between 5–10 years (31/174; 17.8%), 10–15 years (20/174; 11.5%), and 15+ years (26/174; 14.9%). Lastly, 103 respondents held a PhD degree, 12 held an MD degree, and 5 held an MS degree (Supplemental Table 2). Journals most commonly represented in our sample included *Addiction* (33/214; 15.4%), *Psychology of Addictive Behaviors* (31/214; 14.5%), and *Journal of Studies on Alcohol and Drugs* (18/214; 8.4%) (Supplemental Table 3).

### THOUGHTS, ATTITUDES, AND PERCEPTIONS TOWARDS DATA-SHARING

More than one-half of the respondents did not know whether their journal has a data-sharing policy (91/174; 52.3%), nor were they aware if any recent changes have been made to their journal’s data-sharing policies (104/174; 58.0%). When a data-sharing policy was present, most were dictated by the journal’s standards for appropriate data-sharing practices (27/174; 15.5%). Others indicated their journal followed the publisher’s (8/174; 4.6%) or the International Committee of Medical Journal Editor’s (ICMJE) (3/174; 1.7%) data-sharing policies (Supplemental Table 4). Eighty respondents (of 174; 46.0%) indicated their overall perceptions and attitudes toward data-sharing were positive (e.g., the potential benefits of data-sharing outweigh the potential harms). Respondents suggested the optimal timing in which data should be shared were “upon request of the corresponding author” (27/174; 15.5%) and “only after acceptance but before publication” (23/174; 13.2%). Only fourteen respondents suggested all addiction journals should adhere to a universal data-sharing policy. Of those fourteen board members who responded, eight (57.1%) recommended the 2020 NIH Data-Sharing Policy, three (21.4%) recommended the FAIR Guiding Principles for Scientific Data Management and Stewardship, and three (21.4%) recommended the ICMJE Data-Sharing Policy. None of the editorial board members suggested another existing data-sharing policy. Conditions in which respondents believed a third party should be allowed to use to data collected by another researcher were “when the owner and/or funding agencies which provided the original data receives formal acknowledgment in all disseminated work using the original data set” (85/473; 18.0%) and “when the owners of the original data received an opportunity to collaborate on the novel project (e.g., consultation on analytic methods, interpretation/dissemination of results)” (84/473;17.8 %) (Table 1).

On average, board members indicated they “somewhat disagree” that journals should require data-sharing for all manuscripts [M=2.40 (SD=0.08); 95% CI: 2.25–2.56]. In contrast, board members “somewhat agree” that addiction journals should recommend but not require data-sharing for all submitted manuscripts [M=4.12 (SD=0.07); 95% CI: 3.99–4.25] (Table 2). Board members indicated that addiction journals should recommend but not require authors share data stemming from industry-funded studies [M=2.38 (SD=0.04); 95% CI: 2.32–2.47], clinical trials [M=2.26 (SD=0.04); 95% CI: 2.32–2.34], and systematic reviews with meta-analysis [M=2.19 (SD=0.04); 95% CI: 2.11–2.27] (Supplemental Table 5). Potential items that may be incorporated into an ideal data-sharing policy rated as having the highest level of importance included specific instruction on what [M=3.75 (SD=0.12); 95% CI: 3.51–3.99] and how [M=3.56 (SD=0.12); 95% CI: 3.32–3.79] information should be de-identified, how to secure data to prevent malicious use [M=3.48 (SD=0.13); 95% CI: 3.24–3.73], and a detailed account of how electronic data sets should be managed and constructed to facilitate data-sharing [M=3.48 (SD=0.11); 95% CI: 2.92–3.34] (Supplemental Table 6).

### PERCEIVED BENEFITS AND BARRIERS TOWARDS DATA-SHARING

Potential benefits to data-sharing were queried by asking respondents to grade scenarios on level of perceived benefit. Items with the highest ratings were “secondary data use through large scale investigations (e.g., meta-analysis)” [M=3.44 (SD=0.06); 95% CI: 3.31–3.56] and “increase the transparency of research outcomes” [M=3.29 (SD=0.07); 95% CI: 3.14–3.43] (Table 3). Similarly, potential barriers to data-sharing were queried by asking respondents to grade scenarios on level of perceived risk. Items receiving the highest ratings were “lack of metadata standards” [M=3.21 (SD=0.08); 95% CI: 3.06–3.36], “no incentive for the submitting author” [M=3.41 (SD=0.07); 95% CI: 3.27–3.55], “lack of resources” [M=3.53 (SD=0.05); 95% CI: 3.42–3.63], and “protection of privacy (e.g., compliance with HIPAA standards)” [M=3.22 (SD=0.07); 95% CI: 3.07–3.36] (Table 4).

## DISCUSSION

Our survey of editorial board members from top ranking addiction medicine journals indicates that, despite their potentially influential role in directing and implementing policy changes, most board members are unaware of their journal’s current data-sharing requirements. However, the majority of respondents indicated they believe the potential benefits of data-sharing outweigh the potential harms. Although respondents most often voiced their perceptions and attitudes towards data-sharing in a positive light, most indicated they believe addiction journals should recommend but not require authors share their data as a basis for submission. Lastly, responding board members reported secondary data use for large-scale investigations (including meta-analysis) and increased transparency of results and conclusions as potential benefits of data-sharing. In contrast, board members voiced concern for protection of privacy and the burden placed on the submitting author as common barriers towards data-sharing. Here, we expand on the findings of our current investigation and provide commentary to further the conversation surrounding the advantages and disadvantages of data-sharing.

The majority of responding editorial board members indicated they believed the potential benefits of data-sharing outweighed the potential harms. Perceived benefits noted among journal editors included the potential use of data in subsequent meta-analysis, as well as the potential for increased transparency and trust in study outcomes. These sentiments are shared among others in the research community. For example, Deborah Zarin, Director of ClinicalTrials.gov at the National Library of Medicine from 2005–2018, has voiced the benefits of sharing patient-level data to increase the transparency of results from individual clinical trials, as well as to facilitate pooling of results from separate trials to investigate new research questions not pursued in the original trials.^43^ Moreover, previous investigations suggest that meta-analyses performed with de-identified patient-level data, rather than aggregate data, are more likely to detect treatment effects across subgroups, further highlighting the potential benefit of sharing trial data.^44^ Other scenarios in which sharing patient-level data may be beneficial include situations in which clinicians are tasked with interpreting conflicting results from multiple clinical trials. In fact, some authors go as far to suggest that “only through replication can one sort out whether conflicting results are due to chance or true differences”.^45^ In addition to increased transparency and the facilitation of continued research within the field, several moral and ethical arguments in support of data-sharing have been made. For instance, one of the motivating factors for patients participating in clinical research is that their contributions may be used to benefit others.^46–48^ Furthermore, other authors contend that access to relevant data, whether published or unpublished, for inclusion in systematic reviews and meta-analyses may help prevent adverse patient outcomes.^49^ Finally, providing access to trial results stemming from public taxpayer dollars is slowly becoming an expectation among many in the scientific community.^45^ Taken together, the potential benefits of data-sharing are numerous; however, consideration of potential barriers to data-sharing must be taken when determining best practice for sharing clinical trial data.

Respondents to our survey identified poor access to proper resources, lack of standards regarding the interpretation and use of shared trial data, and the protection of patient privacy as key barriers to data-sharing. These barriers, among others, are not uncommon and are discussed in detail in previously published records. For instance, a *BMC Public Health* systematic review identified twenty major barriers to data-sharing which were classified into six broad categories: technical, motivational, economic, political, legal, and ethical.^28^ Avenues to address these obstacles are becoming the focus for many organizations within the research community. For example, the Global Fund—a public health organization which donates upwards of US$4 billion per year towards initiatives focused on defeating AIDS, tuberculosis, and malaria—allocates 5–10% of funds to data collection, monitoring, and evaluation processes.^50^ Initiatives to ensure patients’ right to privacy have also gained traction in recent years while seeking to settle the debate on who the actual “owner” of the data is.^51,52^ More specifically, S4S allows patients to determine when and under what conditions their health data may be released to researchers conducting a study on a particular disease or health condition. Adoption of a similar system throughout the realm of human subjects research may help ease any hesitations towards data sharing that are secondary to patient privacy concerns. Barriers falling into the political, legal, and ethical categories may pose a greater challenge to proponents of data-sharing. Overcoming such challenges would require the creation and widespread implementation of political frameworks including data-sharing agreements and systematic guidelines for sharing clinical trial data. Van Panhuis and colleagues argue this strategy would require a commission aimed at monitoring, mediating, and facilitating data-sharing such that data-sharing practices are “fair, ethical, and efficient”.^28^ Implementing such practices in addiction medicine might be the first step of obtaining the greatest benefits of data-sharing while avoiding some of the associated risks.^28^

The majority of respondents who answered survey questions regarding when data should be shared suggested data should be made available upon request or at the time of acceptance but before publication. This finding suggests editorial board members believe, to some degree, that data-sharing serves some level of importance within the scientific community. Although well intended, previous attempts to “require” data-sharing at the journal level have demonstrated the difficulty of enforcing such policies. For example, Federer et al. published an article which highlights the data-sharing practices of articles published in PLOS journals following the publisher’s policy change which now requires authors to share the data underpinning their results and conclusions.^53^ These authors found that after the publisher’s policy change, author compliance to data-sharing requirements had increased over time, with a steady decrease in the number of publications that did not include a Data Availability statement. Despite this apparent increase in data accessibility, only 20% of the Data Statements found indicated the data were publicly available on a third-party repository (i.e., the preferred and recommended method in the PLOS policy). Others indicated data was available upon request to the corresponding author. Previous studies have shown the success of this method is often variable.^52^ Perhaps adopting stricter policies which require data to be made available at the time of submission or acceptance may help increase the transparency of published results.

An ideal data-sharing policy, if implemented, should adequately address barriers and concerns towards sharing patient-level data, such as those raised among the editors surveyed in our study. Although the “ideal” data-sharing policy is a subject of debate, we recommend addiction journals consider adopting the data-sharing practices outlined by the FAIR Principles. Beginning in 2016, the FAIR principles (Findable, Accessible, Interoperable, and Reusable), a set of principles providing guidance on proper data collection and management strategies, have become increasingly popular within the scientific community. This rise in popularity is a result of the FAIR principles’ ability to increase the overall transparency of research outcomes and better the data stewardship among researchers. When applied properly, the FAIR principles have been shown to optimize the use and reuse of patient level data, as well as to address common concerns regarding the sharing of patient level data.^54,55^ As a result, the expectations placed on research stakeholders—including researchers, journals, funding agencies, and policymakers—to adhere to such guidelines is becoming standard practice.^56^ Furthermore, responses to our survey indicate journal editorial board members believe an ideal data-sharing policy should provide: adequate instruction on what data should be identified and how data should be de-identified; proper security of data to prevent malicious activity; and proper instruction on management of electronic data sets/selection of appropriate data repositories. These concerns, among others, are outlined within the FAIR principles. Therefore, we contend that implementing the FAIR principles within the field of addiction medicine may help address some of the legal, ethical, privacy, and storage concerns voiced by the journal editorial board members within our sample. Such action is particularly important for addiction journals, as previous studies have shed light on the state of data sharing requirements within the field. For instance, Adewumi and colleagues found only 11.5% of studies published in addiction medicine journals included data availability statements, with an even smaller portion providing immediate access to the complete datasets.^57^ Gorman et al. reported that most addiction medicine journals provided some form of data-sharing policy within their author guidelines for submission; however, none required data sharing as a pre-condition for submission or acceptance.^58^ We contend that lax policies that use language such as “recommend” or “suggest” in place of strong language such as “require” will not spark a change in data sharing habits within the field of addiction medicine. Moving forward, journal editorial board members should consider their ability to push for change in their respective journals’ data sharing policies, with the ultimate goal of improving the transparency of the articles they publish.

## LIMITATIONS

Our study has many strengths but is not void of limitations. Regarding strengths, we used a robust methodology which included insight from previously published records on proper survey development and dissemination. We used a thorough approach to accurately identify the names and email addresses for included editorial board members. Lastly, we adhered to our *a priori* protocol when possible, and any deviations we carefully noted in a protocol revision that has been made freely available—along with all study materials and data sets—on the Open Science Framework. Regarding limitations, we did not reach the calculated sample size, and our results should be interpreted accordingly. The extent of missing data necessitating the use of multiple imputation; thus, our results may not reflect the exact perceptions and opinions towards data-sharing among the board members sampled within our study. Furthermore, our study was focused on opinions of editorial board members within the field of addiction medicine and therefore may not be applicable to other fields of medicine, nor may these opinions represent opinions towards data-sharing of other research stakeholders such as funding agencies, researchers, and study participants.

## CONCLUSION

Results from our survey suggest addiction journal editorial board members believe sharing patient-level data has a level of importance within the research community. However, most board members are unaware of their journals’ current data-sharing policies, and the majority believe data-sharing should be recommended but not a requirement for publication within their journals. Future efforts aimed at better understanding common reservations and benefits towards data-sharing, as well as avenues to optimize the sharing of patient-level data while simultaneously minimizing potential risks, are warranted.

## Supplementary Material

Supp. Table 1 Supplemental Table 1. Journal editorial board members’ thoughts, attitudes, and perceptions towards data-sharing
Download: https://www.jospi.org/article/35597-perceptions-and-opinions-towards-data-sharing-a-survey-of-addiction-journal-editorial-board-members/attachment/89847.pdf

Supp. Table 2 Supplemental Table 2. To what degree should journals regulate the data sharing practices of manuscripts published within their journal? (n=174*)
Download: https://www.jospi.org/article/35597-perceptions-and-opinions-towards-data-sharing-a-survey-of-addiction-journal-editorial-board-members/attachment/89981.pdf

Supp. Table 3 Supplemental Table 3. The following statements represent potential benefits to data-sharing rated according to its potential benefit within the addiction medicine literature. (n=174*)
Download: https://www.jospi.org/article/35597-perceptions-and-opinions-towards-data-sharing-a-survey-of-addiction-journal-editorial-board-members/attachment/89982.pdf

Supp. Table 4 Supplemental Table 4. The following statements represent potential barriers to data-sharing ranked according to its potential to hinder or impede data sharing within addiction medicine literature. (n=174*)
Download: https://www.jospi.org/article/35597-perceptions-and-opinions-towards-data-sharing-a-survey-of-addiction-journal-editorial-board-members/attachment/89983.pdf

Supp. Table 5 Supplemental Table 5. What data, if any, should addiction medicine journals require authors to share? (n=174*)
Download: https://www.jospi.org/article/35597-perceptions-and-opinions-towards-data-sharing-a-survey-of-addiction-journal-editorial-board-members/attachment/89984.pdf

Supp. Table 6 Supplemental Table 6. The following statements are examples of specific instructions journals may consider including in their data sharing policy. These instructions may serve to provide guidance on how, when, and where authors should/may share their data. Please rank each statement according to its level of importance in establishing a framework for data sharing that is well understood and easily implemented by authors. (n=174*)
Download: https://www.jospi.org/article/35597-perceptions-and-opinions-towards-data-sharing-a-survey-of-addiction-journal-editorial-board-members/attachment/89985.pdf

## Figures and Tables

**Table 1.** Journal editorial board members’ thoughts, attitudes, and perceptions towards data-sharing

Survey Item	Responses	N (%)
How would you rate your overall thoughts, attitudes, and perceptions of data-sharing? (N=174)	Positive (e.g., the potential benefits of data sharing outweigh potential harms)	80 (46.0)
	Neutral (e.g., indifferent towards data sharing)	21 (12.1)
	Negative (e.g., the potential harms of data sharing outweigh the potential benefits)	12 (6.9)
	Did not respond	61 (35.1)

Has your position as an editorial board member changed your thoughts, attitudes, and perceptions towards data sharing? (N=174)	Yes	11 (6.3)
	No	101 (58.0)
	Did not respond	62 (35.6)

When is the optimal time that authors should share their data? (N=174)	Upon request to the corresponding author	23 (13.2)
	Only after acceptance but before publication	22 (12.6)
	Authors should not be required to share their data	19 (10.9)
	At the time of submission	17 (9.8)
	Within 1 year of publication	16 (9.2)
	Within 6 months of publication	9 (5.2)
	Other	8 (4.6)
	Did not respond	60 (34.5)

Is there a current data-sharing policy that already exists which you believe should be adopted and implemented by all journals in addiction medicine? (N=174)	Yes	11 (6.3)
	No	25 (14.4)
	Unsure	78 (44.8)
	Did not respond	60 (34.5)

If there is a current data sharing policy that already exists which you believe should be adopted and implemented by all journals in addiction medicine, which data-sharing policy would you endorse? (N=14)	National Institute of Health (NIH) Data Sharing Policy (2020)	8 (57.1)
	FAIR principles to data sharing	3 (21.4)
	International Committee for Medical Journal Editors (ICMJE)	3 (21.4)

In your opinion, under what conditions should someone be allowed to use data collected by another researcher? (check all that apply) (N=473)	Owner and/or funding agencies which provided the original data receive formal acknowledgement in all disseminated work using the original data	85 (18.0)
	Owners of the original data receive an opportunity to collaborate on the project (e.g., consultation on analytic methods, interpretation/dissemination of results)	84 (17.8)
	Mutual agreement between the owner of the original data and the party requesting access to the data is reached.	72 (15.2)
	Coauthorship on original manuscript where data was collected	62 (13.1)
	Owner of the original data must receive a list of all products that contain the original data (e.g., educational materials, abstracts, posters, conference presentations, and articles), but approval is NOT required	58 (12.3)
	Owner of the original data receives--at least in part--compensation to cover the cost of collecting, organizing, and maintaining the original data	34 (7.2)
	Owner of the original data must receive a list of all products that contain the original data (e.g., educational materials, abstracts, posters, conference presentations, and articles), AND approval IS required	28 (5.9)
	Legal permission for use of the data is required	28 (5.9)
	Owner of the original data must approve of any/all results based on the original data	22 (4.7)

**Table 2.** To what degree should journals regulate the data sharing practices of manuscripts published within their journal? (n=174*)

Question Items	Mean (SD)**	95% CI
Journals should require data sharing for all manuscripts.	2.40 (0.08)	[2.25–2.56]
Journals should recommend but not require data sharing for all manuscripts.	4.12 (0.07)	[3.99–4.25]
Journals should not require data sharing.	3.05 (0.09)	[2.87–3.23]
Journals should not regulate the sharing of addiction medicine research data.	2.97 (0.09)	[2.79–3.15]

* Includes imputed data to account for missing responses

** Board members were allowed to respond to each question with responses receiving a numerical value according to the following scale: 1=Strongly Disagree, 2=Somewhat Disagree, 3=Neither Agree nor Disagree, 4=Somewhat Agree, 5=Strongly Agree

**Table 3.** The following statements represent potential benefits to data-sharing rated according to its potential benefit within the addiction medicine literature. (n=174*)

Question Items	Mean (SD)**	95% CI
Secondary data use through large scale investigations (e.g., meta-analysis)	3.44 (0.06)	[3.31–3.56]
Secondary data use to answer an independent research question (e.g., not for data synthesis in a meta-analysis)	2.91 (0.06)	[2.78–3.04]
Help reduce the publication of falsified study outcomes	2.96 (0.07)	[2.82–3.11]
Increase the reproducibility of addiction medicine research	3.04 (0.07)	[2.90–3.19]
Increase transparency of research outcomes	3.29 (0.07)	[3.14–3.43]
Verification of research outcomes	3.12 (0.07)	[2.98–3.27]
Increase the public’s trust in clinical trials	2.63 (0.09)	[2.46–2.80]

* Includes imputed data to account for missing responses

** Board members were allowed to respond to each question with responses receiving a numerical value according to the following scale: 1=Not beneficial at all, 2=Slightly beneficial, 3=Fairly beneficial, 4=Extremely beneficial

**Table 4.** The following statements represent potential barriers to data-sharing ranked according to its potential to hinder or impede data sharing within addiction medicine literature. (n=174*)

Technical Barriers		
Question Items	Mean (SD)**	95% CI
Data not preserved or archived.	2.96 (0.08)	[2.80–3.11]
Language - Data not recorded in a local language, limiting the possibility to integrate and use such data together with other data sets, particularly in an international context.	2.68 (0.07)	[2.54–2.82]
Technical issues - lack of software to view or process data.	2.55 (0.08)	[2.39–2.71]
Lack of metadata and standards - lack of information that describes the data, which leads to limited secondary data use.	3.21 (0.08)	[3.05–3.36]
Motivational Barriers		
Question Items	Mean (SD)**	95% CI
No incentives - Data sharing requires time and resources. The benefit of data sharing is often delayed and uncertain (e.g. possibly more efficient disease control programs) instead of immediately relevant to data providers (e.g. scientific credit or training).	3.41 (0.07)	[3.27–3.55]
Opportunity cost - Researchers who have invested time and effort in data collection could anticipate that scientific credit or other opportunities may be lost if data recipients with greater capacity for analysis could gain the majority of credit.	3.22 (0.08)	[3.07–3.38]
Disagreement on data use - Data providers may disagree with the intended secondary use of their data or may consider their data inappropriate for a certain use.	3.12 (0.08)	[2.96–3.27]
Economic or Political Barriers		
Question Items	Mean (SD)**	95% CI
Lack of resources - data sharing oftentimes requires human and technical resources for data preparation, annotation, communication with recipients, computer equipment, internet connectivity, etc.	3.53 (0.05)	[3.42–3.63]
Lack of trust - In the absence of trust, providers could anticipate potential misinterpretation, misuse or intentional abuse of the data.	3.33 (0.05)	[3.22–3.44]
Lack of guidelines - Frequently, official guidelines on data sharing simply do not exist, are unclear or inconsistent.	3.16 (0.07)	[3.03–3.30]
Ownership and copyright - Agencies that collect public health data are often responsible for the protection of individual and community privacy and may feel that a guardianship or ownership role is bestowed on them by the public. Copyright may be used to restrict rather than expand access to data.	3.21 (0.09)	[3.03–3.38]
Ethical Barriers		
Question Items	Mean (SD)**	95% CI
Protection of Privacy - Many agencies have the mandate and authority to collect private data from the population governed by the Health Insurance Portability and Accountability Act (HIPAA) in the US or similar legislation in other countries. A clear distinction between data containing personal identifiers and fully anonymous data may not always be possible, leading to restrictive policies on all types of data due to privacy concerns.	3.22 (0.07)	[3.07–3.36]
Lack of proportionality - The issue of proportionality, the careful deliberation in assessing the risks and benefits that derive from the amount and type of data requested compared to the potential impact of its secondary use, has been identified as a guiding ethical principle for public health data sharing.	2.56 (0.09)	[2.38–2.73]
Lack of reciprocity - Data sharing practices may not always be fair, and data producers have often felt exploited in transactions where they receive little credit or benefit from their work, while data users that can rapidly analyze data and publish results benefit from academic credit and career advancement.	3.05 (0.08)	[2.90–3.21]

* Includes imputed data to account for missing responses

** Board members were allowed to respond to each question with responses receiving a numerical value according to the following scale: 1=Not a barrier at all, 2=Slight barrier, 3=Moderate barrier, 4=Extreme barrier

## Data Availability

Data files used during the completion of this work are available on the Open Science Framework (https://osf.io/63jyz/).
