# Supplementary material for: Perceptions and Opinions Towards Data-Sharing: A Survey of Addiction Journal Editorial Board Members
Source: J Sci Pract Integr. Author manuscript; Available in PMC 2024 May 27. (PMC11129878; doi:10.35122/001c.35597)
Supplement: Supp. Table 1 — Supplemental Table 1. Journal editorial board members’ thoughts, attitudes, and perceptions towards data-sharing Download: https://www.jospi.org/article/35597-perceptions-and-opinions-towards-data-sharing-a-survey-of-addiction-journal-editorial-board-members/attachment/89847.pdf [file NIHMS1994425-supplement-Supp__Table_1.pdf]

Supplemental Table 1. Top 20 Addiction Journals According to Google Scholar Metrics

Addiction
Drug and Alcohol Dependence
Addictive Behaviors
International Journal of Drug Policy
Journal of Behavioral Addictions
Nicotine and Tobacco Research
Alcoholism-Clinical and Experimental Research
International Journal of Mental Health and Addiction
Addiction Biology
Journal of Substance Abuse Treatment
Harm Reduction Journal
Psychology of Addictive Behaviors
Journal of Studies on Alcohol and Drugs
Drug and Alcohol Review
Cannabis and Cannabinoid Research
The American Journal of Drug and Alcohol Abuse
Alcohol and Alcoholism
American Journal on Addictions
Substance Abuse
Journal of Addiction Medicine
