# Supplementary material for: Perceptions and Opinions Towards Data-Sharing: A Survey of Addiction Journal Editorial Board Members
Source: J Sci Pract Integr. Author manuscript; Available in PMC 2024 May 27. (PMC11129878; doi:10.35122/001c.35597)
Supplement: Supp. Table 2 — Supplemental Table 2. To what degree should journals regulate the data sharing practices of manuscripts published within their journal? (n=174*) Download: https://www.jospi.org/article/35597-perceptions-and-opinions-towards-data-sharing-a-survey-of-addiction-journal-editorial-board-members/attachment/89981.pdf [file NIHMS1994425-supplement-Supp__Table_2.pdf]

Supplemental Table 2. Demographic information of the responding journal editorial board members

Survey Item	Response	N (%)
Which of the following represents the gender which you identify with? (N=174)	Male	63 (36.2)
	Female	48 (27.6)
	Prefer not to respond	2 (1.1)
	Did not respond	61 (35.1)
What is your primary role as a member of your journal's respective editorial board? (N=174)	Associate Editor	65 (37.4)
	Board Member (non specified)	15 (8.6)
	Consulting Editor	8 (4.6)
	Peer reviewer	7 (4.0)
	Section Editor	5 (2.9)
	Statistical Editor	4 (2.3)
	Field Editor	3 (1.7)
	Chief Editor	1 (0.6)
	Editor Emeritus	1 (0.6)
	Social Media Editor	1 (0.6)
	Deputy Editor	1 (0.6)
	Senior Editor	1 (0.6)
	No response	62 (35.6)
If you hold another position outside of the journal in which you serve as an editorial board member, which of the following best represents the entity which you are currently employed? (N=174)	Not Applicable	35 (20.1)
	Other	29 (16.7)
	Non-profit	28 (16.1)
	Government	14 (8.0)
	Private/Industry	2 (1.1)
	No response	66 (37.9)
If you hold another position outside of the journal's editorial board, please indicate what type of work you are currently involved in? (select all that apply) (N=230)	Researcher	83 (36.1)
	Professor	61 (26.5)
	Psychologist	14 (6.1)
	Physician (MD, DO)	8 (3.5)
	Not Applicable	2 (0.9)
	No response	62 (27.0)
How many years of service have you worked as an editorial board member? (N=174)	<1	1 (0.6)
	1-3 years	25 (14.4)
	3-5 years	10 (5.7)
	5-10 years	31 (17.8)

	10-15 years	20 (11.5)
	>15	26 (14.9)
	No response	61 (35.1)
Which of the following represents your credentials/highest obtained academic degree (select all that apply)? (N=182)	PhD	103 (56.6)
	MD	12 (6.6)
	MS	5 (2.7)
	BS	3 (1.6)
	MA	1 (0.5)
	BA	1 (0.5)
	ISAM	1 (0.5)
	JD	1 (0.5)
	Board certification equivalent	1 (0.5)
	No response	54 (29.7)
