# Supplementary material for: Perceptions and Opinions Towards Data-Sharing: A Survey of Addiction Journal Editorial Board Members
Source: J Sci Pract Integr. Author manuscript; Available in PMC 2024 May 27. (PMC11129878; doi:10.35122/001c.35597)
Supplement: Supp. Table 3 — Supplemental Table 3. The following statements represent potential benefits to data-sharing rated according to its potential benefit within the addiction medicine literature. (n=174*) Download: https://www.jospi.org/article/35597-perceptions-and-opinions-towards-data-sharing-a-survey-of-addiction-journal-editorial-board-members/attachment/89982.pdf [file NIHMS1994425-supplement-Supp__Table_3.pdf]

**Supplemental Table 3.** Journals represented within cohort of responding addiction medicine journal editorial board members		
**Survey Item**	**Responses**	**N (%)**
For which journal do you belong? (N=214)*	*Addiction*	33 (15.4)
	Psychology of Addictive Behaviors	31 (14.5)
	Journal of Studies on Alcohol and Drugs	18 (10.3)
	Journal of Substance Abuse Treatment	13 (6.1)
	Alcoholism-Clinical and Experimental Research	12 (5.6)
	International Journal of Drug Policy	11 (5.1)
	Drug and Alcohol Dependence	10 (4.7)
	Nicotine and Tobacco Research	10 (4.7)
	Substance Use & Misuse	9 (4.2)
	Drug and Alcohol Review	7 (3.3)
	International Journal of Mental Health and Addiction	7 (3.3)
	Journal of Addiction Medicine	6 (2.8)
	Addictive Behaviors	6 (2.8)
	Addiction Biology	4 (1.9)
	Substance Abuse Journal	4 (1.9)
	Alcohol and Alcoholism	2 (0.9)
	Alcohol Research	2 (0.9)
	Alcohol	2 (0.9)
	Tobacco Control	2 (0.9)
	Journal of Behavioral Addictions	1 (0.5)
	The American Journal on Addictions	1 (0.5)
	Addictive Behavior Reports, International Gambling Studies, Current Addiction Reports	1 (0.5)
	Addictive Disorders & Their Treatment	1 (0.5)
	Alcohol & Alcoholism	1 (0.5)
	Cannabis	1 (0.5)
	Contemporary Drug Problems	1 (0.5)
	Drugs: education prevention and policy	1 (0.5)
	International Gambling Studies	1 (0.5)
	Journal of Psychoactive Drugs	1 (0.5)
	Journal of Addiction and Dependence	1 (0.5)
	Journal of Addictive Diseases	1 (0.5)
	Journal of Behavioral Health Services Research	1 (0.5)
	Journal of Child & Adolescent Substance Abuse	1 (0.5)
	Journal of Concurrent Disorders	1 (0.5)
	Journal of Drug Issues	1 (0.5)

	Journal of Psychoactive Drugs, Addiction Research & Theory, Harm Reduction Journal	1 (0.5)
	Nordic Studies on Alcohol and Drugs, Tobacco Regulatory Science, Smoking Induced Diseases	1 (0.5)

*Includes editorial positions held at one of the top 20 addiction medicine journals included in our sample, addiction medicine journals not ranked in the top 20 according to Google Scholar Metrics, and journals outside of addiction medicine
