# Supplementary material for: Perceptions and Opinions Towards Data-Sharing: A Survey of Addiction Journal Editorial Board Members
Source: J Sci Pract Integr. Author manuscript; Available in PMC 2024 May 27. (PMC11129878; doi:10.35122/001c.35597)
Supplement: Supp. Table 4 — Supplemental Table 4. The following statements represent potential barriers to data-sharing ranked according to its potential to hinder or impede data sharing within addiction medicine literature. (n=174*) Download: https://www.jospi.org/article/35597-perceptions-and-opinions-towards-data-sharing-a-survey-of-addiction-journal-editorial-board-members/attachment/89983.pdf [file NIHMS1994425-supplement-Supp__Table_4.pdf]

Supplemental Table 4. Queries regarding data-sharing policies and practices at journal editorial board members' respective journals

Survey Item	Response	N (%)
Do you currently serve on editorial boards outside of addiction medicine? (N=174)	Yes	68 (39.1)
	No	91 (52.3)
	Did not respond	15 (8.6)
Does your journal currently have a data sharing policy? (N=174)	Yes, as dictated by the journal	27 (15.5)
	Yes, as dictated by the publisher	8 (4.6)
	Yes, as dictated by the ICMJE	3 (1.7)
	No	11 (6.3)
	Unsure	90 (51.7)
	Did not respond	35 (20.1)
Has your journal recently had a change in its data-sharing policy? (N=174)	Yes	7 (4.0)
	No	27 (15.5)
	Unsure	104 (59.8)
	Did not respond	36 (20.7)
If your journal has recently had a change in its data-sharing policy, how recent was this change? (N=174)	Less than 1 year	4 (2.3)
	1-3 years	6 (3.4)
	3-5 years	1 (0.6)
	Not Applicable	114 (65.5)
	Did not respond	49 (28.2)
Did your journal previously have a data sharing policy that has since been discontinued? (N=174)	Yes	1 (0.6)
	No	37 (21.3)
	Unsure	82 (47.1)
	Not Applicable	15 (8.6)
	Did not respond	39 (22.4)
