# Supplementary material for: Perceptions and Opinions Towards Data-Sharing: A Survey of Addiction Journal Editorial Board Members
Source: J Sci Pract Integr. Author manuscript; Available in PMC 2024 May 27. (PMC11129878; doi:10.35122/001c.35597)
Supplement: Supp. Table 5 — Supplemental Table 5. What data, if any, should addiction medicine journals require authors to share? (n=174*) Download: https://www.jospi.org/article/35597-perceptions-and-opinions-towards-data-sharing-a-survey-of-addiction-journal-editorial-board-members/attachment/89984.pdf [file NIHMS1994425-supplement-Supp__Table_5.pdf]

**Supplemental Table 5.** What data, if any, should addiction medicine journals require authors to share? (n=174*)

| Question Items | Mean (SD)** | 95% CI |
|---|---|---|
| Data from clinical trials | 2.26 (0.04) | [2.19-2.34] |
| Data from secondary analysis | 1.96 (0.04) | [1.89-2.03] |
| Data from meta-analysis or systematic reviews | 2.19 (0.04) | [2.11-2.27] |
| Data from observational studies (e.g, cross-sectional studies, cohort studies) | 2.05 (0.03) | [1.98-2.11] |
| Data from animal studies | 2.14 (0.04) | [2.07-2.22] |
| Industry funded studies | 2.38 (0.04) | [2.32-2.47] |
| Funded studies (Any source) | 2.15 (0.04) | [2.08-2.22] |

*Includes imputed data to account for missing responses

**Board members were allowed to respond to each question with responses receiving a numerical value according to the following scale: 1=Should neither be required nor recommended, 2=Should be recommended but not required, 3=Should be required
