# Supplementary material for: Perceptions and Opinions Towards Data-Sharing: A Survey of Addiction Journal Editorial Board Members
Source: J Sci Pract Integr. Author manuscript; Available in PMC 2024 May 27. (PMC11129878; doi:10.35122/001c.35597)
Supplement: Supp. Table 6 — Supplemental Table 6. The following statements are examples of specific instructions journals may consider including in their data sharing policy. These instructions may serve to provide guidance on how, when, and where authors should/may share their data. Please rank each statement according to its level of importance in establishing a framework for data sharing that is well understood and easily implemented by authors. (n=174*) Download: https://www.jospi.org/article/35597-perceptions-and-opinions-towards-data-sharing-a-survey-of-addiction-journal-editorial-board-members/attachment/89985.pdf [file NIHMS1994425-supplement-Supp__Table_6.pdf]

Supplemental Table 6. The following statements are examples of specific instructions journals may consider including in their data sharing policy. These instructions may serve to provide guidance on how, when, and where authors should/may share their data. Please rank each statement according to its level of importance in establishing a framework for data sharing that is well understood and easily implemented by authors. (n=174*)

Question Items	Mean (SD)**	95% CI
Outlines journal's requirements curation of data	2.88 (0.13)	[2.62-3.14]
Outlines journal's requirements for storage of data	3.18 (0.11)	[2.96-3.39]
Provides guidance on study-specific data management and sharing plans	3.09 (0.11)	[2.87-3.31]
Outlines journal's requirements for data repositories	3.30 (0.11)	[3.08-3.53]
Outlines journal's strategy to identify and select appropriate data repository	2.82 (0.12)	[2.59-3.06]
Provides instruction on how electronic data sets should be managed and constructed to facilitate data-sharing	3.21 (0.11)	[2.99-3.43]
Provides instructions on securing data to prevent malicious use of data	3.48 (0.13)	[3.24-3.73]
Provides instruction on what information should be de-identified	3.75 (0.12)	[3.51-3.99]
Provides instruction on how to de-identify data	3.56 (0.12)	[3.32-3.79]

*Includes imputed data to account for missing responses

**Board members were allowed to respond to each question with responses receiving a numerical value according to the following scale: 1=Not important at all, 2=Slightly important, 3=Important, 4=Fairly important, 5=Extremely important
